# Effects of Covid-19 vaccination on different semen parameters

**DOI:** 10.1186/s12610-022-00163-x

**Published:** 2022-08-02

**Authors:** Ziad H. Abd, Samir A. Muter, Ruya Abdulhadi M. Saeed, Omar Ammar

**Affiliations:** 1 College of Medicine, Department of Surgery, University of Anbar, Ramadi, Iraq; 2 College of Medicine, Department of Surgery, University of Baghdad, Baghdad, Iraq; 3 College of Medicine, Department of Community Medicine, University of Anbar, Ramadi, Iraq; 4 Nuffield Department of Women’s & Reproductive Health, Women’s Centre, University of Oxford, John Radcliffe Hospital, Level 3, Oxford, OX3 9DU UK

**Keywords:** COVID-19, Semen analysis, Pfizer-BioNTech mRNA COVID-19 vaccine, Male infertility

## Abstract

**Background:**

The possible effects of COVID-19 vaccines on reproductive health and male fertility in particular have been discussed intensely by the scientific community and the public since their introduction during the pandemic. On news outlets and social media platforms, many claims have been raised regarding the deleterious effects of COVID-19 vaccines on sperm quality without scientific evidence. In response to this emerging conflict, we designed this study to evaluate and assess the effect of the Pfizer-BioNTech mRNA COVID-19 vaccine on male fertility represented by the semen analysis parameters.

**Results:**

Comparing the semen parameters of the participants before and after vaccination, no statistically significant effects on semen volume, pH or normal sperm concentration and morphology were shown. However, there were statistically significant differences on total sperm motility (*P* = 0.05) and progressive motility (*P* = 0.02). These differences are clinically insignificant given the fact that both readings before and after vaccination were within the normal ranges, according to the WHO manual guidelines for the examination and processing of human semen.

**Conclusion:**

Our data suggest that the Pfizer-BioNTech mRNA COVID-19 vaccine has no deleterious effects on semen parameters.

## Résumé

**Contexte:**

Les effets possibles des vaccins contre la COVID-19 sur la santé reproductive et la fertilité masculine, en particulier, ont fait l’objet d’intenses discussions de la part de la communauté scientifique et du public depuis leur introduction pendant la pandémie. Sur les médias et les plateformes de médias sociaux, de nombreuses allégations ont été soulevées concernant les effets délétères des vaccins contre la COVID-19 sur la qualité du sperme, sans preuves scientifiques. En réponse à ce conflit émergent, nous avons conçu cette étude pour évaluer et déterminer l’effet du vaccin COVID-19 à ARNm de Pfizer-BioNTech sur la fertilité masculine représentée par les paramètres d’analyse du sperme.

**Résultats:**

En comparant les paramètres du sperme des participants avant et après la vaccination, aucun effet statistiquement significatif sur le volume de sperme, le pH ou la concentration normale et la morphologie de spermatozoïdes, n’a été montré. Cependant, il y avait des différences statistiquement significatives sur la mobilité totale des spermatozoïdes (*P* = 0,05) et sur leur mobilité progressive (*P* = 0,02). Ces différences sont cliniquement insignifiantes étant donné que les deux lectures avant et après la vaccination se situaient dans des fourchettes normales, selon les directives manuelles de l’OMS pour l’examen et le traitement du sperme humain.

**Conclusion:**

Nos données indiquent que le vaccin à ARNm COVID-19 de Pfizer-BioNTech n’a aucun effet délétère sur les paramètres du sperme.

**Mots-Clés:**

COVID-19, Analyse du Sperme, Vaccin COVID-19 à ARNm de Pfizer-BioNTech, Infertilité masculine

## Background

The 2019 novel coronavirus disease [COVID-19] is an exceedingly transmissible disease of the respiratory tract, first isolated in December 2019 in Wuhan, Hubei Province, China, and then expanding globally. It triggers a severe acute respiratory syndrome named coronavirus disease 2019 (COVID-19) [1]. In March 2020, the World Health Organization (WHO) announced COVID-19 to be a global pandemic. Over the following two years, COVID-19 affected millions of people across the world, with a high mortality rate and a lack of a defined treatment protocol [1–3]. The disease affected various aspects of life, including but not limited to social, economic and health aspects [4]. Clinically, COVID-19 symptoms ranged from asymptomatic or mild upper respiratory tract symptoms to severe and fatal respiratory distress syndrome. It has also been reported to affect other organ systems, mainly the gastrointestinal tract [5].

The possible effect of COVID-19 on reproductive health and especially male fertility has been a point of discussion by the scientific community and the public since the start of the pandemic. It was suggested that SARS-CoV-2 affects male fertility relies on its ability to cross the blood-testis barrier and its binding to the angiotensin-converting enzyme 2 (ACE2) in Leydig cells [6–8]. Others have suggested that SARS-CoV-2 affects spermatogenesis via an immune-mediated type of orchitis or the disease-associated inflammatory response [9].

As in many other viral diseases, in response to the COVID-19 pandemic, different types of vaccines were made, aiming at minimising disease severity, and in turn its adverse social and economic effects. Pfizer-BioNTech mRNA COVID-19 vaccine received the U.S. Food and Drug Administration (FDA) approval on August 23, 2021, for individuals ages 16 years and older [10]. Pfizer-BioNTech mRNA COVID-19 vaccine is mainly composed of messenger ribonucleic acid (mRNA), lipids, salts, and sugars [10].

The list of approved vaccines has been extended to include many other internationally and locally accepted vaccines such as Moderna, Janssen and AstraZeneca. In Iraq, the first vaccine that gained local authorities’ approval and was licensed for use was the Sinopharm vaccine, followed by Pfizer-BioNTech and then AstraZeneca. However, most of the vaccinated Iraqis have received the Pfizer-BioNTech mRNA COVID-19 vaccine, mainly related to the availability of this vaccine possible by the US government donations and Iraqi Ministry of Health Contracts.

Since the start of the COVID-19 pandemic, there was a great deal of debate and discussion on the possible effects of COVID-19 on male fertility. This discussion has been extrapolated to the use of different COVID-19 vaccines. The fear of adverse effects of the COVID-19 vaccine on male fertility has always been a reason for vaccination hesitancy among the public [11]. Recent reports have explored the effects of mRNA COVID-19 vaccines on male infertility [4, 12, 13]. In two small prospective cohort studies of healthy men, Gonzalez et al. [12] and Lifshitz et al. [14] did not observe a reduction in semen parameters up to 90 days after COVID-10 mRNA vaccination. However, these studies enrolled only a small number of young and healthy men from the United States and Israel. In order to provide more data on the matter of COVID-19 vaccines and male infertility, we designed this study to evaluate and estimate the safety of the Pfizer-BioNTech mRNA COVID-19 vaccine on male fertility as represented by the semen parameters in a sample of Iraqi men. We believe more data is needed regarding the topic from different countries and patient cohorts.

## Material and methods

This is a two-centre prospective observational study carried out at Ar-Razzi IVF Centre, Ramadi, Iraq, and Al-Rayyan IVF Centre in Baghdad, Iraq. After obtaining ethical approvals and written consent from all participants, 60 adult men younger than 50 years were enrolled. Men were partners of women attending IVF clinics experiencing infertility due to clear female factors such as tubal factor infertility or ovulatory disorders. Partners of these women being treated in these centres are regularly reassessed by a semen analysis on a six-monthly basis to detect and treat any emerging male factor. All semen analysis results are archived on protected computers. Those who visited the centres with their wives for regular follow-up and treatment and had received two COVID-19 vaccine doses were offered to have their wives’ visit fees waived if they agreed to participate in the study and provide a semen sample for semen analysis. All participants gave written consent and were given the right to withdraw from the study whenever they wanted with no consequences.

Inclusion criteria were healthy adult males from 18 to 50 years old with previous normal semen analysis results and had had the second covid-19 vaccine at least 90 days before the new semen analysis test.

After 2–4 days of abstinence, a single semen sample was obtained through masturbation and collected in sterile jars for analysis. The assessment started after the liquefaction of semen. Parameters studied included (semen volume, liquefaction time, pH, sperm concentration, sperm motility, and sperm morphology) and were compared to the volunteers’ pre-vaccination results. All samples were analyzed within 30–60 min of ejaculation. Semen samples were analyzed manually strictly following the WHO manual guidelines for the examination and processing of human semen [15]. Semen analysis was performed by the same andrologist in each centre to avoid performer variability. All samples were then discarded properly, and analysis results were archived into the volunteers’ files.

Exclusion criteria included men who were diagnosed with COVID-19 disease, or other chronic diseases, started any long-term medication or started smoking after their last semen analysis. Those who reported an extended post-vaccination fever of more than three days were also excluded.

Covid-19 vaccines were administered strictly in governmental health centres, and people who received the vaccine were provided with a “vaccination card” stating the dates of the first and second doses and the type of vaccine administered. Additionally, participants were asked about the side effects they experienced after each dose.

For participants with more than one result available in our data system, the more recent available semen analysis results before vaccination were used to compare with the post-vaccination semen analysis.

Medians and IQRs were reported for all different semen analysis parameters of pre-and post-vaccination semen samples. The Wilcoxon-matched pairs signed-rank test was used to compare pre-and post-vaccination semen parameters. A JAMOVI project (2021) version 1.8.1.0 computer software was used. A *P* < 0.05 was considered statistically significant.

## Results

Participants’ mean age was 37.2 years with an average body mass index of 26.4, and one-third of them were smokers, as shown in Table 1. All smokers have been smoking for many years before the study and none have reported a major change in their habits in terms of the daily number or type of cigarettes smoked.

**Table 1:** Participant demographics data

Demographics	Value
Number of participants	60
Age, (mean ± SD, median; range)	37.2 ± 5.3, 36; 25–45
Body mass index (kg/m2] [mean ± SD, median; range)	26.4 ± 6.2, 25.8; 21.5–37.3
Smoking	
YES	20 (33.3%)
NO	40 (66.6%)

Semen parameters were compared between participants before and after vaccination. The mean number of days between the pre-vaccine semen analysis and the first vaccination dose was 100.5 ± 69. The mean number of days between the first and second doses was 101 ± 37 and 133 ± 39, respectively. Vaccination did not affect semen volume, pH, sperm concentration, or morphology. Nevertheless, there were statistically significant differences in total sperm motility (*P* = 0.05) and progressive motility (*P* = 0.02). The details of sperm parameters before and after vaccination are presented in Table 2.

**Table 2:** Effects of COVID-19 vaccination on different semen parameters

Semen Analysis Parameters	Normal Value according to WHO [15]	Median [IQR]		*p*-value
		Pre-vaccination	Post-vaccination	
No. of Participants		60	60	
Volume, mL	> 1.5	3.5 (2.4)	3.3 (2.4)	0.63
pH	> 7.2	8 (0)	8 (0)	0.52
Sperm concentration, mill/mL	> 15	74 (43)	70 (42)	**0.63**
Total motility, %	> 40	74 (22.8)	69 (16.8)	**0.05**
Progressive motility (A + B) %	> 32%	60 (19.3)	59 (16.5)	**0.02**
Normal morphology, %	> 4%	5 (1.25)	5 (1.25)	0.33

Wilcoxon matched pairs signed rank-test was used to compare between the different parameters

## Discussion

Several studies have explored the possible effects of the disease on fertility and semen parameters; more recently, researchers are focusing on the possible effects of the COVID-19 vaccine, especially after the mass vaccination campaigns across the globe.

Six months after the beginning of the pandemic, different vaccines were approved to be used and numerous reports regarding the vaccine's effects on male fertility were published. Our study aimed to estimate the effect of the Pfizer-BioNTech mRNA COVID-19 vaccine on semen parameters as an indicator of male fertility. Our results demonstrated a slight reduction in both total motility and progressive motility after the Pfizer-BioNTech mRNA COVID-19 vaccine. The difference in these parameters before and after vaccination was clinically insignificant and both samples were within the normal ranges according to the WHO manual guidelines for the examination and processing of human semen [15]. The particularity with semen analysis is that we expect variations between the same individual giving two samples regardless of any treatment or intervention. There are also factors related to laboratory staff performance and the collection of the whole ejaculate by participants. There were no significant effects on the other parameters such as semen volume, pH or sperm concentration morphology. Our data are in agreement with previous reports that reported no significant deleterious effects of mRNA COVID-19 vaccines on semen parameters [12–14, 16]. Barda et al. [16] found that total sperm count and total motile count increased after the second vaccine compared to samples before vaccination in a cohort of 33 healthy donors. Sperm motility did not change after the vaccine [16]. In Lifshitz et al. [14] study, none of the vaccinated men produced abnormal results apart from two participants (2 out of 75), one oligozoospermic, and one with reduced motility. In a study by Gonzalez et al. [12], they found that sperm parameters did not differ after the two doses of COVID-19 mRNA vaccine administration.

## Conclusions

Our data suggest that the Pfizer-BioNTech mRNA COVID-19 vaccine has no deleterious effects on semen parameters. However, a larger sample size with stricter inclusion criteria, such as including only men who had previous children through natural pregnancy, will be beneficial for future studies. Using a computerised system for the semen analysis will help in reducing the performer estimate errors.

## Data Availability

All data are available from the authors on reasonable request. N/A.

## Abbreviations

ACE2: Angiotensin-converting enzyme 2
FDA: Food and Drug Administration
WHO: World Health Organization
